# Lower Plasma Creatinine and Urine Albumin in Individuals at Increased Risk of Type 2 Diabetes with Factor V Leiden Mutation

**DOI:** 10.1155/2014/530830

**Published:** 2014-03-04

**Authors:** Andreas Peter, Andreas Fritsche, Fausto Machicao, Peter P. Nawroth, Hans-Ulrich Häring, Berend Isermann

**Affiliations:** ^1^Department of Internal Medicine, Division of Endocrinology, Diabetology, Angiology, Nephrology and Clinical Chemistry, Eberhard-Karls University Tübingen, Otfried-Müller-Straße 10, 72076 Tübingen, Germany; ^2^Institute for Diabetes Research and Metabolic Diseases of the Helmholtz Center Munich, University of Tübingen, 72076 Tübingen, Germany; ^3^German Center for Diabetes Research (DZD), 85764 Neuherberg, Germany; ^4^Department of Internal Medicine I and Clinical Chemistry, University of Heidelberg, Im Neuenheimer Feld 410, 69120 Heidelberg, Germany; ^5^Department of Clinical Chemistry and Pathobiochemistry, Otto-von-Guericke-University Magdeburg, Leipziger Straße 44, 39120 Magdeburg, Germany

## Abstract

The factor V Leiden (FVL) mutation is the most frequent genetic cause of venous thrombosis in Caucasians. However, protective effects have been suggested to balance the disadvantages. We have recently observed protective effects of FVL mutation on experimental diabetic nephropathy in mice as well as an association with reduced albuminuria in two human cohorts of diabetic patients. In the present study we aimed to reevaluate these findings in an independent, larger cohort of 1905 Caucasians at risk of developing type 2 diabetes and extend possible associations to earlier disease stages of nephropathy. Carriers of FVL mutation had a significantly lower urine albumin excretion (*P* = 0.03) and tended to have lower plasma creatinine concentrations (*P* = 0.07). The difference in plasma creatinine concentrations was significant after adjustment for the influencing factors: age, gender, and lean body mass (*P* = 0.048). These observations at a very early “disease” stage are an important extension of previous findings and suggest that modification of glomerular dysfunction by FVL mutation is relevant during very early stages of diabetic nephropathy. This makes the underlying mechanism an interesting therapeutic target and raises the question whether FVL mutation may also exert protective effects in other glomerulopathies.

## 1. Introduction

The factor V Leiden (FVL) mutation is the most frequent genetic cause of venous thrombosis in Caucasians. The high frequency of the FVL mutation implies associated positive effects which balance the negative sequels of the FVL mutation [1]. Indeed, several protective effects associated with the FVL mutation and potentially conveying a positive selection bias have been proposed [2]. Thus, the presence of the FVL mutation has been associated with lower peripartal blood loss and reduced mortality in sepsis [2, 3]. Obviously the amelioration of these acute and potentially deadly health threats mediates a positive selection pressure, maintaining the FVL mutation in the gene pool despite the potentially negative effects associated with venous thrombosis. We have recently proposed that the protective mechanism(s) selected in FVL carriers may not only be relevant in acute diseases, but also modulate the course of chronic diseases, which pose the prevailing health threats in modern times. Thus, low but sustained thrombin generation associated with the presence of the FVL mutation results in larger but more stable plaques in atherosclerosis prone mice, indicating a partial benefit [4]. Furthermore, in a second mouse model we observed a protective effect of the FVL mutation on experimental diabetic nephropathy [5]. The potential mechanisms include prevention of glomerular endothelial cell and podocyte dysfunction as well as reduction of extracellular matrix accumulation [5]. These results were supported by an association of FVL mutation with reduced albuminuria in two human cohorts of 200 type 1 and 350 type 2 diabetic patients [5]. In the present study we aimed to reevaluate these findings in an independent, larger cohort and extend the possible associations to earlier disease stages of nephropathy.

## 2. Materials and Methods

We investigated 1905 Caucasians who participated in ongoing studies on the pathogenesis of type 2 diabetes [6]. A written consent was obtained from all participants and the local ethics committee approved the study protocol. Unlike in the previous report, which included patients with established diabetes mellitus, inclusion criterion for the current study was an elevated risk of developing type 2 diabetes, that is, family history of type 2 diabetes, body mass index (BMI) > 27 kg/m^2^, diagnosis of impaired glucose tolerance, or previous diagnosis of gestational diabetes. All participants underwent an oral glucose tolerance test (OGTT). After an overnight fast, 75 g glucose was ingested at 8:00 a.m.; plasma glucose and insulin concentrations were determined after 0, 30, 60, 90, and 120 min. Insulin sensitivity was assessed from glucose and insulin values during the five-point OGTT with the Matsuda index [7]. In 800 subjects urine samples were available for albumin measurements.

The HPLC method (Tosoh A1c 2.2 HLC-723, Tokyo, Japan, and Tosoh G8 HPLC A1c analyzer) was used for HbA1c measurements, plasma glucose was determined using a bedside glucose analyzer (glucose-oxidase method; YSI, Yellow Springs Instruments), urine albumin was determined on the BN Prospec nephelometer, plasma creatinine was measured enzymatically on the ADVIA 1800 clinical chemistry analyzer, and insulin was analyzed using the ADVIA Centaur XP immunoassay system (all from Siemens Healthcare Diagnostics, Eschborn, Germany). Lean body mass was measured by bioelectrical impedance (BIA-101; RJL Systems, Detroit). The Sequenom MassARRAY platform (Sequenom, San Diego) was used to genotype for FVL mutation (rs6025). For statistical analyses nonnormally distributed parameters were log transformed. Statistical analyses were conducted with JMP 8.0 (SAS Institute, Cary, NC).

## 3. Results and Discussion

Participant characteristics are supplied in Table 1. Based on a 75 g oral glucose tolerance test and/or HbA1c measurements performed at inclusion, 105 subjects were newly diagnosed with diabetes according to the criteria of the American Diabetes Association [8]. The frequency of newly diagnosed diabetes mellitus did not differ between FVL-negative and -positive individuals. To evaluate the impact of the FVL mutation on albuminuria and plasma creatinine a dominant model was used since only one homozygous FVL carrier was identified.

FVL carriers showed no significant differences in metabolic traits or blood pressure (Table 1). Carriers of FVL mutation had a significantly lower urine albumin excretion (*P* = 0.03) (Table 1). Even when the subjects with newly diagnosed diabetes were excluded, this trend was still present (*P* = 0.050). We next analyzed whether the FVL mutation also influences plasma creatinine levels. Carriers of FVL mutation tended to have lower plasma creatinine concentrations (*P* = 0.07). The difference in plasma creatinine concentrations was significant after adjustment for the influencing factors: age, gender, and lean body mass (*P* = 0.048). This association remained unchanged after exclusion of subjects with newly diagnosed diabetes (*P* = 0.048). The lower urine albumin excretion in individuals with an increased risk to develop type 2 diabetes underscores that FVL mutation conveys a protective effect in regard to urine albumin excretion, an established marker of glomerular dysfunction. In addition to lower urine albumin excretion we observed lower plasma creatinine values in FVL-positive individuals. Of note, a retrospective analysis of our previously published cohort of type 2 diabetic patients [5] revealed a similar trend for lower plasma creatinine levels (mean 1.10 mg/dl in FVL-negative patients versus 0.89 mg/dl in FVL-positive patients, *P* = 0.082). The impact of the FVL mutation appears to differ in regard to urine albumin excretion and plasma creatinine concentrations, considering that the difference in plasma creatinine concentrations was only of borderline significance or showed a trend between FVL-negative and FVL-positive individuals, while urine albumin excretion differed significantly in these two studies. However, the differences in plasma creatinine concentrations may be less obvious, since albuminuria is an early manifestation of diabetic nephropathy, preceding the rise in the plasma creatinine and as renal impairment will not progress in all patients with albuminuria to more advanced stages hallmarked by increased creatinine plasma concentrations [9]. The future evaluation of a larger number of diabetic patients with advanced stages of diabetic nephropathy may provide more robust results in regard to the effect of the FVL mutation on plasma creatinine concentration in type 2 diabetic patients.

Intriguingly, in the present study we even observed a lower urine albumin excretion as well as plasma creatinine concentrations in nondiabetic individuals at risk of Developing type 2 diabetes and hence at a very early “disease” stage. This is an important extension of previous findings and indicates that the underlying mechanism through which the FVL mutation modifies glomerular dysfunction is of functional relevance during very early stages of diabetic nephropathy, making the underlying mechanism an interesting therapeutic target. Hence, further studies evaluating the nephroprotective mechanism associated with the FVL mutation in diabetic patients are warranted. In addition, these studies raise the question whether the FVL mutation may exert protective effect in other glomerulopathies. Providing answers to these questions may identify novel therapeutic targets for diabetic nephropathy and potentially other glomerular diseases.

## Figures and Tables

**Table 1:** Participant characteristics of the total cohort and those subjects with available urine samples are displayed.

Characteristic	All subjects		*P*	Subjects with urine samples		*P*
	Wildtype	FVL mutation		Wildtype	FVL mutation	
*N*	1780	125	—	744	56	—
Gender (females/males)	1171/609	84/41	0.75*	481/263	41/15	0.19*
Age (years)	39.7 ± 0.3	41.1 ± 1.2	0.26	43.7 ± 0.5	44.4 ± 1.7	0.72
Diabetes	99 (5.6%)	6 (4.8%)	0.71*	57 (7.7%)	3 (5.4%)	0.53*
Body weight (kg)	87.7 ± 0.6	85.7 ± 2.4	0.41	99.3 ± 1.12	95.6 ± 4.10	0.38
BMI (kg·m^−2^)	30.17 ± 0.21	29.73 ± 0.81	0.61	34.30 ± 0.37	33.36 ± 1.37	0.50
Lean body mass (kg)	57.02 ± 0.33	55.63 ± 1.27	0.29	60.09 ± 0.60	57.84 ± 2.19	0.32
HbA1c (%)	5.42 ± 0.01	5.42 ± 0.05	0.88	5.69 ± 0.02	5.59 ± 0.09	0.26
Blood pressure syst. (mmHg)	123.9 ± 0.49	126.4 ± 1.84	0.19	125.8 ± 0.6	125.9 ± 2.18	0.94
Blood pressure diast. (mmHg)	77.8 ± 0.33	78.6 ± 1.24	0.55	79.4 ± 0.4	79.5 ± 1.44	0.96
Insulin sensitivity_OGTT_ (AU)	14.94 ± 0.25	14.64 ± 0.95	0.76	11.48 ± 0.34	11.87 ± 1.29	0.77
Fasting glucose (mmol/L)	5.20 ± 0.02	5.26 ± 0.06	0.34	5.39 ± 0.02	5.27 ± 0.09	0.19
Plasma creatinine (mg/dL)	0.849 ± 0.004	0.822 ± 0.014	0.07	0.856 ± 0.006	0.825 ± 0.024	0.24
Urine albumin (mg/L)	—	—	—	31.3 ± 7.6	10.7 ± 27.6	**0.03**
Plasma creatinine (mg/dL)**	0.849 ± 0.003	0.823 ± 0.013	0.048**	—	—	—

Values represent means ± SE (standard error). For statistical analyses, nonnormally distributed parameters were log transformed. ISI: insulin sensitivity index. Significant differences are marked by bold font.

*Chi-square test; **adjusted for age, gender, and lean body mass.
